# Cryo-EM structure of the complex between transglutaminase 2 and the 45 kDa domain of fibronectin

**DOI:** 10.1016/j.jbc.2026.113326

**Published:** 2026-07-13

**Authors:** Julie Elisabeth Heggelund, Ahmad Ali-Ahmad, Marie Kongshaug Johannesen, Nikolina Sekulić, Ludvig M. Sollid

**Affiliations:** 1 Norwegian Coeliac Disease Research Centre, Institute of Clinical Medicine, University of Oslo, Oslo, Norway; 2 Department of Immunology, Oslo University Hospital-Rikshospitalet, Oslo, Norway; 3 Department of Molecular Medicine, Institute of Basic Medical Sciences, University of Oslo, Oslo, Norway

**Keywords:** complex, cryo-electron microscopy, fibronectin, protein–protein interaction, transglutaminase

## Abstract

Fibronectin (FN) and transglutaminase 2 (TG2) engage in a high-affinity interaction. This interaction affects anchoring, migration and survival of cells, and high cellular expression of TG2 is associated with metastasis potential of cancers. Previous work has mapped the interaction to involve the N-terminal part of TG2 and an N-terminally located 45 kDa domain of FN (FN45). In particular, the I_8_ module of FN45 is reported to be necessary and sufficient for TG2 binding. Here, we present the cryo-EM structure of a complex between human TG2 and FN45 at 2.8 Å resolution. In the structure, the distal region of the TG2 N-terminal domain binds the FN45 I_8_ module, whereas the proximal N-terminal region and adjacent catalytic core segment bind the FN45 I_7_ module and, to a lesser extent, the FN45 II_2_ module. The FN45 I_7_ and FN45 I_8_ modules have about equal buried surface areas in their interaction with TG2. A glycine residue at position 127 of TG2 is crucial for interaction with Trp553 of the FN45 I_8_ module. Mutation of this residue to isoleucine, which is carried by transglutaminase 3 that does not bind FN45, abrogates the binding of FN45 to TG2. This finding suggests that the interaction of the FN45 I_7_ module with TG2 is contingent on the FN45 I_8_ module making an interaction with TG2. Further, this finding highlights the region surrounding residue 127 of TG2 as a promising target for structure-based design of anticancer drugs aimed at disrupting the TG2–FN interaction.

Transglutaminase 2 (TG2) is a ubiquitously expressed enzyme that catalyzes modification (*i*.*e*. transamidation or deamidation) of glutamine residues in polypeptides in a Ca^2+^- and sequence-dependent fashion (1). The enzyme has four domains, an N-terminal β-sandwich domain, a catalytic core domain and two C-terminal β-barrel domains (C1 and C2) (Fig. 1*A*). In the presence of GTP/GDP that serves as an allosteric inhibitor the enzyme takes a “closed” conformation (2) whereas on binding irreversible inhibitors at the active site it takes an “open” conformation with the two C-terminal β-barrel domains swinging 120° away from the catalytic core domain (3). A structure of a Ca^2+^-bound intermediate state was recently reported (4). TG2 is produced on free ribosomes in the cytosol and is chiefly localized in the cytosol and the nucleus, yet the protein is also found outside of cells bound to extracellular matrix components (1). One such component, likely a collagen fibrillar structure, binds to the C2 domain of TG2 (5). Another component, whose interaction with TG2 has been extensively studied, is fibronectin (FN) (6, 7, 8). FN is a glycosylated homodimer of 440 kDa where each protomer of FN consists of three types of repeating units (*i*.*e*. module types I, II and III), with a total of 12 type I modules, 2 type II modules and 15 to 17 type III modules (Fig. 1*B*) (9). FN is involved in assembly of extracellular matrix as it serves as a scaffold for connection of various extracellular matrix components (10). Apart from TG2, FN binding partners include denatured collagen (gelatin) and isolated collagen chains (11, 12), proteoglycans/glycosaminoglycans including heparin/heparan sulfate (13), tenascin (14) and thrombospondin-1 (15).

**Figure 1:**
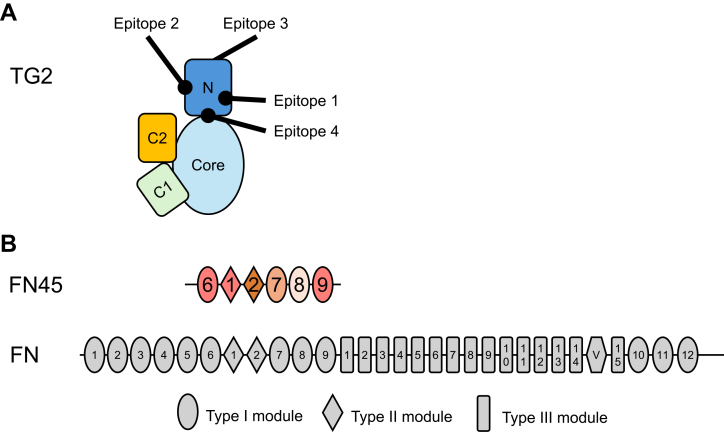
**Schematic representations of TG2 and FN.***A*, TG2 has four domains, an N-terminal β-sandwich domain, a catalytic core domain and two C-terminal β-barrel domains (C1 and C2). The localization of epitopes recognized by autoantibodies of celiac disease patients is indicated. *B*, FN is a dimer where each protomer consists of three types of repeating modules (*i*.*e*. type I, II and III) as indicated. Indicated is also the composition of the FN45 fragment which is also known as the gelatin-binding domain.

The interaction between extracellular matrix FN and cell-surface bound TG2 affects anchoring and migration of cells (16, 17). Moreover, FN-mediated interactions with TG2 influence cell survival (18, 19, 20), and in multiple cancer types, high cellular expression of TG2 is associated with metastasis potential (20, 21, 22). Thus, disruption of the FN–TG2 interaction is considered a promising therapeutic strategy, and small-molecule inhibitors have been developed to target this interaction (23, 24). Blockade of the TG2–FN interaction by such small-molecule inhibitors has been shown, among other effects, to exert *in vivo* activity in a model of cancer cell dissemination (24).

The main binding of FN to TG2 is mediated by an N-terminally positioned 45 kDa domain (FN45; composed of modules I_6_ II_1,2_ I_7,8,9_) as demonstrated by the equal binding of this domain and full-length FN to TG2 (25). A truncated 15 kDa fragment that encompasses modules I_7,8,9_ was found to behave similarly to the whole 45kDa fragment regarding cell adhesion, spreading and migration, whereas a fragment representing the I_8_ module alone was found to be necessary and sufficient for binding to TG2 *in vitro* (26). Of note, FN45 is involved in the binding of gelatin, and this fragment is also known as the gelatin-binding domain (12). As to the engagements on the TG2-side of the interaction, early work suggested that the interaction is mediated by the N-terminal domain of TG2 (8). Hang and coworkers reported that two β-strands in this domain are involved in the binding to FN45, with key roles of D94 and D97, as mutation of these two residues to alanine led to significantly impaired binding (27). TG2 is targeted by highly disease-specific autoantibodies in celiac disease (28, 29), and the epitopes of these autoantibodies are primarily located in the N-terminal region of the protein (30). Using monoclonal antibodies (mAbs) cloned from single plasma cells of celiac disease lesions (29), Cardoso and coworkers probed the binding of TG2 to FN45 (31, 32). It was found that the mAbs that are specific for epitope 1 and epitope 4 but no other epitope-specific mAbs (30), compete with FN45 binding to TG2 (31). Furthermore, it was found that residues K30, R116 and H134, which are part of epitope 1 (33), are involved in the interaction of TG2 with FN45 (31). Subsequent analysis reported that binding of TG2 to FN is independent of TG2 having an “open” or “closed” conformation, that the two C-terminal domains of TG2 are not involved in the binding and that a chimeric TG2 molecule with the N-terminal domain coming from transglutaminase 3 (TG3) failed to bind FN45 (32). Hydrogen-deuterium exchange analysis revealed decreased deuterium uptake in fragments containing residues 41 to 58, 120 to 129 and 130 to 135, suggesting that these regions are involved in the interaction with FN (32).

These previous studies provide important insights into the interaction between TG2 and FN, yet structural information is pivotal. While a couple of computational models have been reported on the FN45–TG2 interaction (26, 34), currently no experimentally obtained structural data are available. This lack of structural data hinders deeper mechanistic insight and prevents the structure-based design of molecules that block the FN–TG2 interaction. We aimed to fill this knowledge gap by solving the structure of the FN45-TG2 complex. Here we report the cryo-EM structure at 2.8 Å resolution of the full-length human TG2 complexed with human FN45. Based on the structure, variants of TG2 were produced and tested for binding to FN45 by ELISA and surface plasmon resonance (SPR). The results suggest a dynamic model for the interaction of TG2 with the I_7_ and I_8_ modules of FN45, where the I_8_ module seems to govern the interaction.

## Results

### Preparation of TG2-FN45 complex

The 45 kDa proteolytic fragment of fibronectin (FN45) was derived from human plasma provided by a commercial supplier (Fig. S1*A*). LC-MS/MS analysis of the predominant band obtained by SDS-PAGE and subsequent in-gel digestion revealed the presence of residues 291 to 592 representing modules I_6_ II_1,2_ I_7,8,9_ of the canonical 45 kDa fragment with a sequence coverage of 68% (Fig. S2 and Table S1). Three peptides of the I_1_ and I_2_ modules were also present, but at low intensity, suggesting incomplete purification of the fragment. Human TG2 was recombinantly produced in insect cells and purified by His-tag affinity chromatography (Fig. S1*B*). The TG2 and FN45 complex was formed by incubating purified TG2 with FN45 in excess (Fig. S1*C*). Gel filtration demonstrated a monodisperse peak with a small peak for excess FN45, indicating complete complex formation (Fig. S1*D*).

### Cryo-electron microscopy structure of TG2-FN45 complex

**Figure 2:**
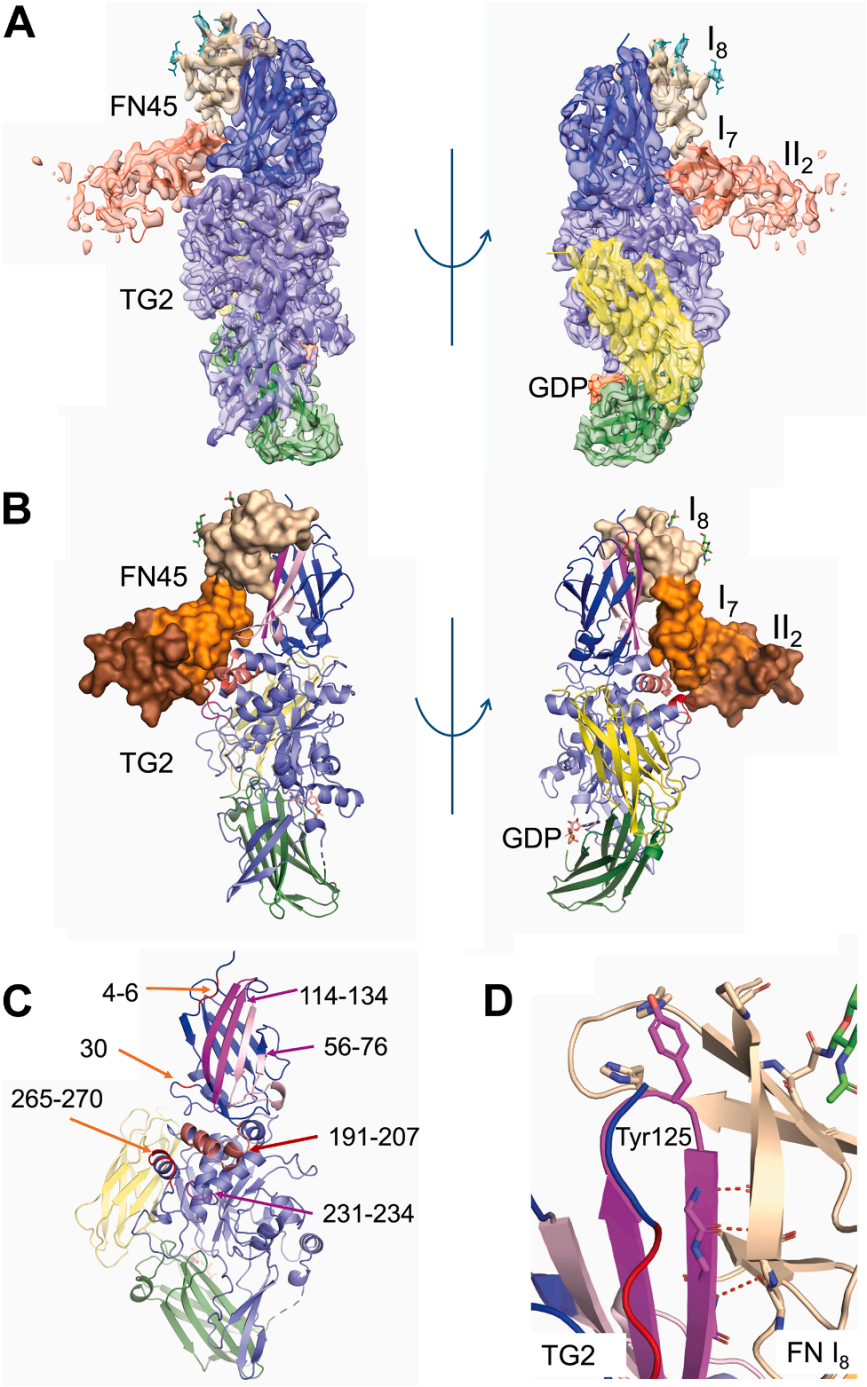
**The cryo-EM structure of TG2 binding to FN45.** TG2 is shown in cartoon representation with the N-terminal domain in *blue*, the core domain in *light blue*, the C1 domain in *green* and the C2 domain in *yellow*. GDP is shown as sticks. Glycosylation of FN45 is shown as *green* sticks. *A*, the cryo-EM structure shown with density. *B*, interaction of TG2 with FN45. FN45 is shown in surface representation with the I_8_ module in wheat, the I_7_ module in *orange* and the II_2_ module in *brown*. Areas of TG2 buried by FN45 are colored in *pink* and *red*. *C*, areas of TG2 buried by FN45. *D*, close-up of interactions of TG2 with FN45 involving residues 125 to 129 in TG2 and 551 to 555 in FN45, showing backbone H-bonds as *red dashed lines*. Both molecules are shown in cartoon representation, with selected side chains shown as sticks. TG2 is colored in the same pattern as in the other panels, and the fibronectin I_8_ module is colored in wheat.

The cryo-EM structure of TG2 in complex with FN45 was solved to 2.8 Å resolution and reveals one molecule of TG2 binding to the central parts of one FN45 molecule (Table 1 and Figs. 2, S3, *A–E* and S4). The overall structure of TG2 is very similar to previously published crystal structures of the closed conformation of TG2 with no Ca^2+^ bound (PDB IDs: 1KV3, 4PYG, 6A8P), with an r.m.sd of 1.219 Å relative to 6A8P. Even though no GDP was added during protein purification, we observed distinct density for a GDP molecule bound in the GDP pocket between the core domain and the C1 domain. Conceivably, this GDP originated from the cytosol of insect cells where the recombinant TG2 was expressed. No density was observed for the N-terminal sequence extension containing a His_6_-tag and a biotinylated Avi-tag, indicating that this region does not form stable interactions with any part of the TG2–FN45 complex. In the structure of the FN45 domain, the modules I_8_ and I_7_ were modeled into a clear cryo-EM map, while the density for module II_2_ was comparatively weaker. In contrast, the N-terminal modules (II_1_, I_6_) and the C-terminal module I_9_ were not modeled as the map was poorly defined in these regions. This lack of density likely reflects conformational flexibility, as evidenced by the local resolution map (Fig. S4).

**Table 1:** Cryo-EM data collection and processing statistics

Magnification	130,000 x
Voltage (kV)	300
Electron exposure (e/Å^2^)	57.5
Defocus range (μm)	−0.6 to−1.8 (0.2)
Pixel size (Å)	0.65
Deposited data	EMD-58048, PDB: 30UC
Initial reference map	*ab initio*
Symmetry imposed	C1
Initial particle images (no.)	726,000
Final particle images (no.)	238,000
Box size (Å)	273
Pixel size (Å)	0.65
Map sharpening B-factor	−67
Map resolution (Å), FSC threshold	2.80
Initial model used	AlphaFold3 (TG2 + I_8_)
Model resolution (Å), FSC threshold	2.80
Model composition:	
Non-hydrogen atoms	6521
Protein residues	810
Ligands	GDP: 1, NAG: 2
Water	54
B-factors:	
Protein	74.24
N-linked glycosylation	115.99
GDP	66.27
Water	56.07
r.m.s. deviations: bond lengths (Å)	0.002
r.m.s. deviations: bond angles (°)	0.415
Molprobity score	1.18
Clash score	3.93
Rotamer outliers (%)	0
Ramachandran plot profile, favored (%)	98.25
Ramachandran plot profile, allowed (%)	1.75
Ramachandran plot profile, disallowed (%)	0.00
Q score: All	0.491
Q score: Chain A (TG2)	0.501
Q score: Chain B (FN45)	0.438
Model vs Data:	
CC (mask)	0.75
CC (box)	0.62
CC (peaks)	0.57
CC (volume)	0.73
Mean CC for ligands	0.45

Analysis of the TG2-FN45 complex with PDBePISA reveals a total buried surface area on FN45 of 1443 Å^2^ comprising 685 Å^2^ from the I_8_ module, 655 Å^2^ from the I_7_ module and 114 Å^2^ from the module II_2_ (Table S2). The buried surface residues are given in Table S3, and Table S4 gives an overview of H-bonds and salt bridges between TG2 and FN45. The complex has an overall calculated solvation free energy gain (ΔG_solv_) of −9.0 kcal/mol, with the FN45 I_7_ module clearly contributing most of this gain in solvation free energy. Notably, the I_8_ module has more hydrophilic interactions with TG2 than the I_7_ module (Table S2).

**Figure 3:**
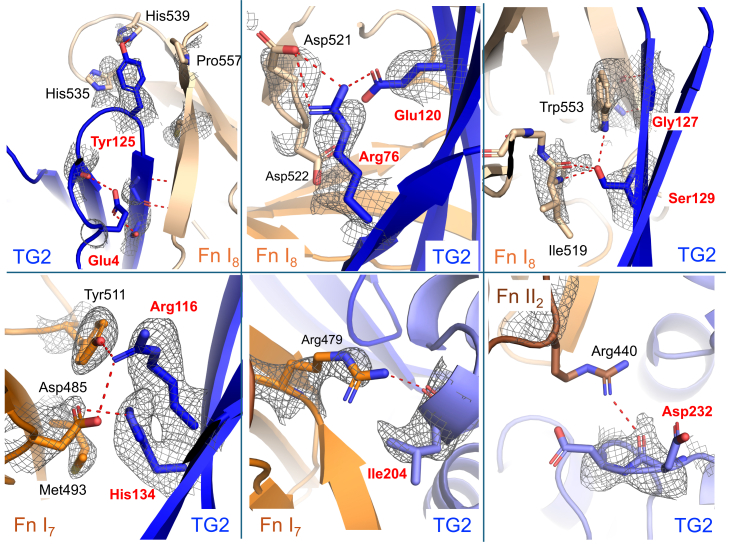
**Close-ups of interactions of key residues involved in the binding between TG2 and FN45.** TG2 and FN45 are shown in cartoon representation with residues shown in sticks. The N-terminal domain of TG2 is colored *blue*, and the core domain is colored *light blue*. FN45 module I_8_ is colored wheat, I_7_ is colored *orange* and II_2_ is colored *brown*. The residues of focus in each panel are highlighted with *red* labels, H-bonds are shown as *red* dashed lines. The cryo-EM map carved around the side chains of residues of interest is shown in *grey* mesh, except in the panel for Ser129 and Asp232 where the map is carved around entire residues.

In the interaction interphase between TG2 and FN45, the I_8_ module wraps around the distal part of the N-terminal domain of TG2, and the I_7_ module binds to the proximal part of the N-terminal domain and the adjacent part of the core domain of TG2 (Fig. 2). These two FN45 modules bury TG2 β-strands comprising residues 114 to 134, 56 to 76, as well as 191 to 207. The II_2_ module is in an area with poor density and interacts at a small area where Arg440 in its most likely side chain rotamer forms an H-bond to Gln232 of TG2. Some of the most notable TG2 residues that form electrostatic interactions with FN45 are Arg76, Arg116, Glu120, Tyr125, Gly127, Ser129, His134 and Leu204. Depictions of the interactions of these residues, with corresponding density, are given in Figure 3. The stretch 127 to 129 in TG2 has main-chain interactions with 551 to 553 of I_8,_ resembling an interchain β-sheet. Glu4 H-bonds to Ser128 and Thr126 of TG2, potentially stabilizing this significant area of TG2. Tyr125 is sandwiched between His535 and Pro557, likely with a pi-pi interaction with His535 as well as an H-bond to His539. Arg76 forms a salt bridge to Asp521, while Glu120 H-bonds to Arg76, possibly orienting this residue for interaction with the negatively charged 521 to 522 loop in I_8_. Arg116 forms a salt bridge to Asp485 and an H-bond to Tyr511. His134 is in close proximity to Met493 of the I_7_ module, likely forming an aromatic-methionine interaction as well as a salt bridge to Asp485. The carbonyl of Leu204 forms an H-bond with Arg479 of the I_7_ module.

We also generated a model of the TG2 FN45 interaction using the AlphaFold3 server. This model positions the I_8_ module in almost perfect alignment with the I_8_ module of the cryo-EM structure, but with no further interactions between TG2 and FN45 (Fig. S5).

### Functional testing by ELISA of interacting residues in TG2

**Figure 4:**
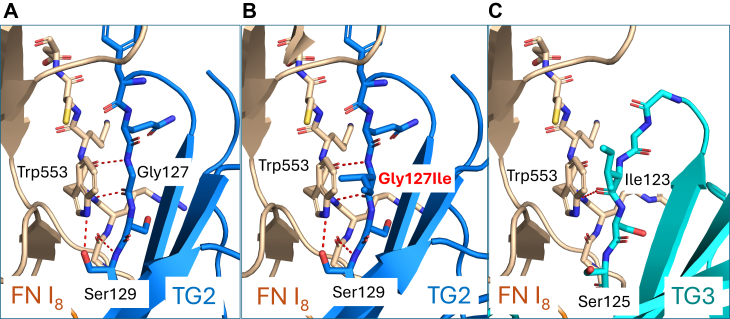
**The impact of residue Gly127 in TG2 for binding of FN45.***A*, The binding of residues 125 to 129 in TG2 with 551 to 556 in FN. *B*, the mutation of Gly127 to Ile in TG2 causes a clash with Trp553 of FN45. The isoleucine is introduced by mutagenesis in PyMol. *C*, overlay of TG3 (PDB ID: 8OXW) showing the close proximity of Ile123 in TG3 with Trp553 in FN. All molecules are shown in cartoon representation with residues of interest in sticks. TG2 is colored *blue*, TG3 is colored cyan and FN45 is colored wheat. H-bonds are depicted as *red dashed lines*.

**Figure 5:**
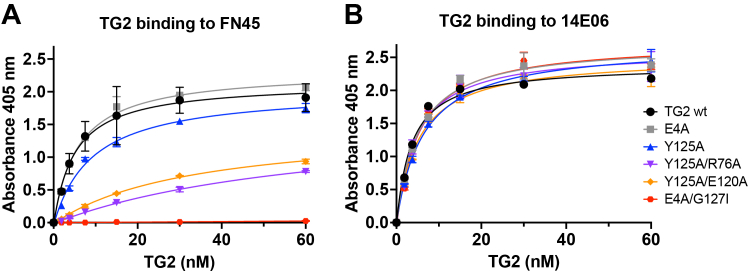
**Binding of TG2 variants to FN45 in ELISA.** Increasing concentrations of TG2 variants were tested for binding to either (*A*) FN45 immobilized to the ELISA plate, or (*B*) anti-TG2 antibody 679-14-E06 immobilized to the ELISA plate. The experiment was done in duplicate, repeated twice. The graphs are showing the mean ( ± standard deviation) of one representative experiment.

Because the chimeric TG2 molecule containing the N-terminal domain of TG3 does not bind FN45 (32), we compared the cryo-EM structure of TG2 with the structure of TG3 (PDB ID: 8OXW) (35). The tight binding of Gly127 of TG2 to Trp553 of FN45 (Figs. 4, *A*–*C*, S6) would not be possible for TG3, as this transglutaminase carries an isoleucine residue at the corresponding position (Ile123 in TG3). It is also clear from mutagenesis analysis of this TG2 residue in PyMol that the interchain β-sheet seen in the cryo-EM structure would be disrupted (Fig. 4*B*). To investigate what impact a glycine to isoleucine substitution would have on binding to FN45, we created a TG2 variant harboring this mutation and tested it for binding to FN45 by ELISA. The G127I mutation was introduced together with E4A and showed complete abrogation of binding to FN45 in the ELISA setup (Fig. 5). A TG2 E4A variant showed only a small reduction in binding, pointing to the isoleucine clash being the essential feature for the loss of binding of the E4A/G127I variant.

After investigating the cryo-EM structure both manually and by PDBePISA, we identified several other residues of TG2 that had not previously been investigated. We produced and tested a number of these as TG2 variants for binding to FN45. All these variants had the C277A mutation to prevent aberrant cross-linking activity during purification and assays, as well as a His_6_-tag for easy purification and detection. By coating microtiter plates with FN45, incubating the TG2 variants and detecting with an anti-His tag antibody, we could show that both double variants Y125A/R76A and Y125A/E120A had a clear reduction of binding, with Y125A/R76A having the most pronounced effect. Testing single variant Y125A showed only a slight reduction in binding, pointing to the salt bridge of Arg76 being important (Fig. 3). Glu120 might be important in the positioning of Arg76, but it was also predicted to H-bond to the main chain of Asp521 and Asp522 in FN I_8_ by PDBePISA (Table S4). The TG2 variants were also tested in a separate plate for binding to anti-TG2 mAb 679-14-E06 which recognizes epitope 1 of TG2 which is a conformational epitope located in the N-terminal part of TG2 (30) (Fig. 1*A*). All variants were recognized by this mAb to an equivalent extent (Fig. 5), indicating that the mutated residues are not part of the antibody epitope, that the proteins are all properly folded and intact and of similar concentration in the ELISA experiment.

### SPR analysis of binding between TG2 variants to FN45 and FN

**Figure 6:**
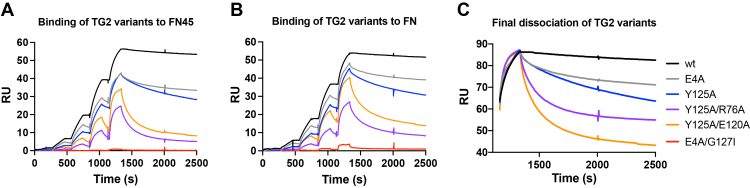
**SPR analysis of binding of TG2 variants to FN45 and FN.***A*, representative sensorgrams of the binding of TG2 variants to FN45. *B*, representative sensorgrams of the binding of TG2 variants to FN. *C*, Overlay of the final dissociation curves to show the difference in dissociation rates. The FN45 experiment was performed four times for each TG2 variant, while the FN experiment was performed twice for each TG2 variant.

The binding of TG2 variants to FN45 was additionally investigated using SPR with FN45 immobilized on the chip and TG2 variants injected in increasing concentration using a single-cycle kinetics setup. To determine the kinetic parameters, the sensorgrams were fitted using both a 1:1 binding model and a two-state binding model (Fig. S7). For the TG2 variants, the two-state model was selected as it captured a rapid initial dissociation phase observed in the sensorgrams that could not be accommodated by the 1:1 model. Conversely, the TG2 wt protein did not exhibit this quick initial dissociation, so to avoid overparameterization, the standard 1:1 binding model was retained for the wt protein. Sensorgrams and kinetic values are shown in Figures. 6, S8 and Table 2.

**Table 2:** SPR kinetic values

FN45					
TG2 analyte	ka (R1: 10^4^ Ms^−1^, R2: 10^−4^ s^−1^)	kd (10^−3^ s^−1^)	R_max_ (RU)	Chi^2^ (range)	K_D_ (nM)
wt	9.70 ± 0.45	0.029 ± 0.007	55–97	1.9–5.7	0.30 ± 0.08
E4A					
R 1	15.5 ± 1.36	5.89 ± 0.67	41–67	0.6–2.1	1.33 ± 0.11
R 2	61.8 ± 2.41	0.223 ± 0.013			
Y125A					
R 1	8.51 ± 0.76	2.22 ± 0.67	44–68	0.3–1.0	3.94 ± 0.09
R 2	36.4 ± 7.21	0.659 ± 0.020			
Y125A/R76A					
R 1	7.39 ± 0.56	7.68 ± 0.41	31–51	0.1–0.4	37.5 ± 2.18
R 2	11.2 ± 0.28	0.631 ± 0.055			
Y125A/E120A					
R 1	11.8 ± 1.17	7.89 ± 0.74	40–62	0.6–2.2	22.7 ± 2.94
R 2	12.0 ± 0.54	0.623 ± 0.135			
E4A/G127I					
R 1	N/D	N/D	N/D	N/D	N/D
R 2	N/D	N/D			

FN					

wt	8.52 ± 0.38	0.025 ± 0.005	58 ± 7	1.6–1.9	0.30 ± 0.08
E4A					
R 1	11.8 ± 0.36	6.45 ± 0.13	42 ± 1	0.6–0.8	1.40 ± 0.09
R 2	82.7 ± 4.68	0.217 ± 0.00			
Y125A					
R 1	6.70 ± 0.16	1.77 ± 0.12	47 ± 0	0.3	4.06 ± 0.13
R 2	31.5 ± 1.17	0.574 ± 0.013			
Y125A/R76A					
R 1	6.87 ± 0.69	6.96 ± 0.09	33 ± 1	0.2–0.3	24.7 ± 1.84
R 2	19.2 ± 0.40	0.615 ± 0.002			
Y125A/E120A					
R 1	9.93 ± 0.34	6.30 ± 0.36	44 ± 2	2.1	14.5 ± 1.68
R 2	18.9 ± 1.52	0.562 ± 0.112			
E4A/G127I					
R 1	N/D	N/D	N/D	N/D	N/D
R 2	N/D	N/D			

ka: association rate constant, kd: dissociation rate constant, K_D_: binding affinity, R 1: binding reaction 1, R 2: binding reaction 2. N/D: not determinable. R_max_ values are listed as ranges for FN45 since two separate flow channels with different ligand densities were used.

The two-state binding model is consistent with an induced fit where TG2 docks onto one FN45 module first, then locks into place upon binding of another FN45 module. The binding of the TG2 variants was consistent with the ELISA data, with decreasing binding strengths in this order: wt > E4A > Y125A > Y125A/E120A > Y125A/R76A > E4A/G127I. All these variants have substitutions in residues interacting with the I_8_ module, thus possibly slowing the initial docking reaction driven by electrostatic interactions. Their reduced ka1 and increased kd1 as compared to the wt TG2 ka- and kd values are in keeping with this interpretation. The TG2 variant E4A/G127I showed a small increase in signal for the two highest concentrations of TG2 (64 nM and 256 nM), leading to a repeat experiment testing even higher concentrations (up to 10 μm) (Fig. S8), but still we were unable to calculate a K_D_ accurately for this variant.

## Discussion

The cryo-EM structure of the TG2-FN45 complex reveals that TG2 engages the FN45 domain through an interface that extends longitudinally along one of its two β-sheets of the N-terminal domain. The distal part of this TG2 domain makes contacts with the I_8_ module, the proximal part of the domain and the adjacent part of the core domain interact with the I_7_ module, whereas a small part of the core domain contacts the II_2_ module. In the interaction with TG2, the I_7_ module and the I_8_ module have approximately the same size of their buried surface areas.

A striking finding is that despite the extensive interaction between TG2 and the I_7_ module in the cryo-EM structure, the TG2 G127I mutation almost completely abolishes the binding of TG2 to FN45. Residue 127 of TG2 contacts the Trp553 of the I_8_ module, suggesting that the I_8_ module mechanistically governs the interaction of FN45 with TG2. In general, a two-state model gave the best fit for the SPR sensorgrams assessing binding of TG2 to FN45. This finding is compatible with an initial binding of TG2 to the I_8_ module followed by an involvement of the I_7_ module after a conformational change. Interestingly, the model of the FN45 and TG2 interaction generated by the AlphaFold server positions the I_8_ module as in the cryo-EM structure, but with the I_7_ and I_8_ modules placed in a straight line and with no interaction by the I_7_ module. In the cryo-EM structure, the angle between the two modules is 60° with additional TG2 contacts by the FN45 I_7_ module. This binding dynamic would explain why the G127I mutation can almost abolish TG2 binding to FN45.

The obtained structure provides an opportunity to reconsider experimental results probing the interactions between TG2 and FN45. Mutations of residues D94 and D97 in TG2 were reported to impair binding to FN45 (27). This finding was not confirmed in a subsequent study (31). As both residues are located far from the interaction site, it is difficult to rationalize how they would influence binding. Results obtained by hydrogen-deuterium exchange are largely in keeping with the structure as the two TG2 fragments that had the most pronounced decrease in deuterium uptake (fragments 120–129 and 130–135) (32) both have many residues engaged in the interaction. Observations suggesting relatedness of epitope 1 of celiac disease autoantibodies with the binding site of FN45 (31, 32), also align well with the cryo-EM structure. TG2 residues K30, H134, and R116, which were found to be part of epitope 1 (33) and which by mutational analysis were indicated to engage in the binding to FN45 (31, 32), all make contact in the structure with the I_7_ module. The 2000-fold lower binding observed for TG2 variant K30E/R116A/H134A (32) suggests that the I_7_ module contributes substantially to the binding of intact FN45 to TG2 with the I_8_ module being present (26). The results of Soluri *et al*. (26), who studied binding to TG2 by FN fragments expressed as FN–Fc constructs, are consistent with the structure in showing that the I_8_ module binds TG2. Their finding that the I_7_ module alone has no detectable binding is consistent with the model that the interaction of this module is contingent on the I_8_ module making an initial interaction with TG2.

The observations of this study that TG2 variants with alterations R76A, Y125A and G127I all have impaired binding to FN45 yet are recognized unimpaired by the epitope 1–specific mAb 679-14-E06, suggest that the binding site of FN45 for TG2 is overlapping but not identical to the epitope footprint of celiac disease epitope 1 autoantibodies. Definitive insights on this issue await structures of complexes between Fab fragments of epitope 1 antibodies bound to TG2.

The interaction between FN and TG2 is considered a promising therapeutic target, and small-molecule inhibitors have been identified from high-throughput screening of a compound library and by the making of second-generation analogs (23, 24). Second-generation analogs were shown in cell cultures to cause membrane ruffling, to give a delay in the formation of stable focal contacts and mature adhesion points, and to disrupt the organization of the actin cytoskeleton (24). Further, in an *in vivo* model that measures intraperitoneal dissemination, the small molecule inhibitors inhibited the adhesion of ovarian cancer cells to the peritoneum. With the structure of the complex between FN45 and TG2 reported herein, structure-based design of drugs can now be undertaken. As the G127I mutation almost abolishes binding of TG2 to FN45, the space around the G127 residue appears particularly promising to explore in such an endeavor. The G127 residue is in close contact with Trp553 of FN45, whose side chain is partly embedded in a shallow binding groove on the surface of TG2 (Fig. 6). This cavity could potentially be targeted by a small molecule inhibitor.

Modules of FN45 are known to interact with extracellular matrix components other than TG2. A fragment representing FN modules I_8_-I_9_ was shown to bind two distinct but sequence-similar peptides from the α1 chain of collagen type I (36, 37). In crystal structures of the corresponding complexes, the same β-sheet (residues 550–555) of module I_8_ that is engaged in the binding to TG2 is involved in binding of the collagen peptides, with a particular involvement of Trp553. Moreover, module I_7_ has also been demonstrated to interact with collagen peptides (38). Potentially, such interactions could compete with and influence the binding between TG2 and FN45.

The previously published computer models of the docking of FN to TG2 (26, 34) are at variance with this cryo-EM structure. The model presented by Soluri *et al*. (26) placed the interaction of the I_8_ module with TG2 residues K30, R116 and H134. These TG2 residues rather interact with the I_7_ module in the cryo-EM structure. Selcuk *et al*. (34) presented two models involving the I_7-9_ modules of FN45. One model (PDB ID: 9A3Z) shows binding of the I_7_ and II_2_ modules to the core domain of TG2, but no binding to the I_8_ module, while another model (PDB ID: 9A3Y) places the FN45 I_7_ module in contact with the TG2 N-terminal domain, but in a different location and in the opposite orientation compared to the cryo-EM structure. None of the interactions is the same as the ones we observe in the experimental structure. The model generated by the AlphaFold3 server positions the I_8_ module in perfect alignment with this module of the cryo-EM structure, but it fails to predict the interaction of the remaining FN45 modules with TG2. Overall, these results speak to the notion that in the age of computer modeling of molecules, experimentally established structures are still very important to obtain.

## Experimental procedures

### Mass spectrometry analysis of FN45

For ascertainment of sequence and coverage of FN modules in the commercially obtained FN45 (Sigma-Aldrich; F0162, lot 281077), the protein was analyzed by LC-MS/MS. FN45 was mixed with 4x Laemmli loading buffer containing 5% β-mercaptoethanol and heated to 95 °C for 5 min. Subsequently, 2.25, 4.5 and 0.6 μg were loaded into separate wells on a 4 to 20% TGX SDS-PAGE gel and electrophoresed at 180V for 35 min. The gel was then stained with InstantBlue Coomassie protein stain (Abcam ISB1L). The middle gel band (4.5 μg) was excised using a clean scalpel and subjected to in-gel reduction, alkylation and digestion (39). The resulting peptides were analyzed by EVOSEP liquid chromatography system coupled to a timsTOF fleX mass spectrometer (Bruker Daltonics) *via* a CaptiveSpray ion source. The standard 30SPD method from EVOSEP was used to separate the peptides on a 15 cm reversed-phase C18 column (EV-1137). The raw file was submitted to MaxQuant software version 2.7.0.0 (40) for protein identification.

### Preparation of recombinant TG2 in insect cells

Recombinant human TG2 was produced in Sf + insect cells with an N-terminal His_6_-tag followed by an Avi-tag that was biotinylated within the insect cells by co-expression of BirA biotin ligase. Cell pellets were resuspended in lysis buffer: 50 mM sodium phosphate pH 8.0, 300 mM NaCl, 10 mM imidazole, 1% NP-40 substitute and EDTA-free cOmplete protease inhibitor cocktail (Roche, Basel, Switzerland) and incubated for 1 h on ice with vortexing every 15 min. The suspension was centrifuged at 18,000*g* for 40 min, and the supernatant was collected. TG2 was purified by Ni-NTA chromatography in a gravity column setup. The Ni-NTA resin was washed with 50 mM sodium phosphate pH 8.0, 300 mM NaCl, 10 mM imidazole and eluted with the same buffer with 200 mM imidazole. The eluted protein was dialyzed against storage buffer 20 mM Tris-HCl, pH 7.4, 300 mM NaCl, 1 mM DTT, 1 mM EDTA. The final protein was aliquoted and frozen at −80 °C, in a concentration of 3.6 mg/ml.

### Analysis of FN45 and TG2 before complex formation

For SDS-PAGE analysis, 2 μg of TG2 and FN45 were each mixed with 4x Laemmli loading buffer containing 10% β-mercaptoethanol and heated at 95 °C for 5 min. The samples were added to a 12% TGX SDS-PAGE gel and run at 180V for 40 min. The gel was subsequently stained with InstantBlue Coomassie protein stain, and the resulting bands were compared with a molecular weight marker (Precision Plus Protein Dual Color Standard (Bio-Rad, 1,610,374)).

### Preparation of TG2-FN45 complex

TG2 was mixed with FN45 in a molar ratio of 1:1.1 TG2:FN45 in buffer 20 mM Tris-HCl, pH 7.4, 300 mM NaCl, 1 mM DTT, 1 mM EDTA. The complex was incubated at 21 °C for 2 h before it was subjected to size-exclusion chromatography using a Superdex 200 Increase 10/300 column (Cytiva) at 0.8 ml/min using the same buffer. A single peak fraction was kept on ice and brought to the grid preparation area the same day.

### Cryo-EM grid preparation, data acquisition and processing

The TG2-FN45 complex sample was prepared by diluting the purified protein to a final concentration of 0.35 to 0.7 mg/ml in the same buffer as that was used for gel filtration. This sample was then applied to freshly glow-discharged Quantifoil R2/1300 mesh copper grids (Quantifoil Micro Tools GmBH, Grosslöbichau). The grids were blotted for 5 s and vitrified by plunging into liquid ethane using an FEI Vitrobot automatic plunge freezer (Thermo Fisher), with the environmental chamber maintained at 100% humidity throughout the process. Data collection was carried out at the Cryo-EM Swedish National Facility (SciLifeLab) using a Titan Krios transmission electron microscope (Thermo Fisher Scientific). The microscope operated at an accelerating voltage of 300 kV and was equipped with a Field Emission Gun (FEG) source. The workflow of image processing and 3D construction of cryo-EM images is depicted in Figure S1, *A–E*. Micrographs were recorded in bright-field mode using flood-beam illumination and a Gatan K3 direct electron detector. The detector was operated in super-resolution counting mode at a nominal magnification of 1,300,00x, corresponding to a calibrated physical pixel size of 0.65 Å. Automated data acquisition was performed using EPU software, with a defocus range of 600 nm to 1800 nm applied in 200 nm steps. Each movie was composed of 50 frames with a total average electron dose of 57.5 e^−^/Å^2^. Image processing was performed entirely within CryoSparc V4.4.1 (41). Movie frames were aligned using patch motion, CTF was estimated in a patch manner, and several hundreds of particles were manually picked. The resulting useful particles were then used for automatic particle picking using template picking. A total of 2.8 million particles were picked using template picking, followed by several rounds of 2D classification. After *ab initio* reconstruction, 726 k particles were retained for further 3D classification and local refinement using a local mask generated in ChimeraX (42). A final set of 204,524 particles was selected for the final local refinement. The global resolution of the map reached 2.80 Å based on the gold-standard Fourier shell correlation (FSC) 0.143 cut-off criterion, with half-maps generated through totally independent refinement. Euler angle distribution of particles used in the 3D reconstruction was visualized in ChimeraX. All maps were filtered to local resolution using CryoSPARC 4.4.1 with a B-factor determined in the refinement step. Directional FSC analysis confirmed a high degree of sampling isotropy with a sphericity value of 0.812. The local resolution of the structure is shown in Figure S4.

### Model building of TG2-FN45 complex

The model was built in Coot (43) using crystal structures of TG2 (PDB ID: 6A8P) and models of individual modules of FN45 generated with the AlphaFold3 server (https://alphafoldserver.com/) (44). The N-linked glycosylations at residues Asn528 and Asn542 in the I_8_ module assisted in the placement of FN45, since there was visible density for the glycans. Only one N-acetylglucosamine was added to the model. The model was carefully assessed using Molprobity (45) and refined using Phenix real_space_refine (46). The figures were prepared with PyMol and ChimeraX (42). The evaluation of the molecular interactions between TG2 and FN45 was done with PDBePISA (https://www.ebi.ac.uk/pdbe/pisa/) (47). To assess the accuracy of the model generated by the AlphaFold3 server, this prediction model was compared with the final cryo-EM structure of the TG2-FN45 complex.

### Preparation of TG2 variants in *E*. *coli*

Synthetic genes for the recombinant human TG2-C277A and variants were ordered from GenScript (Piscataway, NJ, USA) in a pET28a + vector with a His_6_-tag added. All variants had a C277A mutation to remove any chances of enzymatic activity. The vector was transformed into *E*. *coli* BL21 (DE3) cells (Thermo Fisher Scientific). A single colony was inoculated into a pre-culture of 5 ml LB + kanamycin (30 μg/ml) and grown for 3.5 h at 37 °C, 210 rpm shaking. The pre-culture was added to a 1 L culture of LB + kanamycin (30 μg/ml) in a 3 L baffled shaker flask. When the culture reached OD_600nm_ of 0.5 to 0.7, the temperature was lowered to 20 °C, and the protein expression was induced by adding 0.2 mM IPTG. Low temperature was necessary to avoid inclusion body formation. The cultures were left to produce overnight for 16 h before they were harvested by centrifugation. The cells were lysed by sonication in the presence of lysozyme in 50 mM sodium phosphate pH 8.0, 300 mM NaCl, 10 mM imidazole, 0.1 mg/ml lysozyme and EDTA-free complete protease inhibitor cocktail (Roche, Basel, Switzerland). GDP (50 μm) was added to the lysis buffer to promote a “closed” conformation of TG2 since the C277S variant of TG2 has been shown to mainly be in the “open” conformation, thereby potentially leading to non-covalent multimerization of TG2 (48). TG2 was purified by Ni-NTA chromatography in a gravity column setup. The Ni-NTA resin was washed with 50 mM sodium phosphate pH 8.0, 300 mM NaCl, 10 mM imidazole and eluted with the same buffer with 200 mM imidazole. The eluted protein was dialyzed against storage buffer: 20 mM Tris-HCl, pH 7.4, 300 mM NaCl, 1 mM DTT, 1 mM EDTA. The final protein was aliquoted and frozen at −20 °C, in concentrations of 0.5 to 2.2 mg/ml. A typical yield from this protocol was 3 to 7 mg per liter of *E*. *coli* culture.

### Binding of TG2 variants to FN45 assessed by ELISA

Solutions of anti-TG2 antibody 679-14-E06 (10 μg/ml) (30), produced as human IgG1 as described (29), and FN45 (2 μg/ml) were prepared in PBS, and 100 μl of each solution was applied to separate wells of a Maxisorp plate before incubation at 21 °C for 1 h. The plates were washed with PBS + 0.05% Tween (PBS-T) using a Tecan Hydroflex plate washer before they were blocked by incubation with 5% dry milk (Merck, Darmstadt, Germany; 115,363) for 1 h. TG2 was serially diluted in PBS-T starting with a concentration of 60 nM, and 100 μl of each concentration was applied to the plate in duplicate and incubated for 1 h. After removing the non-binding protein, TG2 was detected using anti-His antibody (SouthernBiotech; 4603–04) conjugated to alkaline phosphatase. Bound TG2 was measured as an increase in absorbance at 405 nm, and the values were plotted using a three-parameter non-linear regression in GraphPad Prism (GraphPad Software, Boston, MA, USA).

### Binding of TG2 variants to FN45 assessed by surface plasmon resonance (SPR)

The SPR experiments were run on a T200 Biacore instrument (Cytiva) using Biacore T200 control software version 2.01. All runs were performed in running buffer PBS + 0.005% surfactant P20 (Cytiva, BR100054), at 25 °C, keeping the sample chamber at 8 °C. FN45 was diluted to 2.5 μg/ml in 10 mM sodium acetate pH4.5 and was immobilized using amine coupling in two channels (immobilization rates: 62 RU and 80 RU) of a CM5 Series S chip (Cytiva, 29,104,988). FN full-length (Sigma-Aldrich, 341,635) was diluted to 10 μg/ml and immobilized in 10 mM sodium acetate pH4.5 using amine coupling in one channel (immobilization rate: 331 RU). One flow channel was treated with mock immobilization and used as a reference channel. The experiments were run at a flow rate of 70 μl/min. Inactive TG2 variants (C277A) were injected over the chip using the single cycle method with five different concentrations: 1 nM, 4 nM, 16 nM and 256 nM, preceded by five injections of buffer. The chip was regenerated at the end of each cycle with an injection of 10 mM NaOH for 30 s. Four separate single-cycle experiments were performed for each variant of FN45, and two separate single cycle experiments were performed for the full-length FN. The experiment with the E4A/G127I variant of TG2 was repeated with higher concentrations: 39 nM, 156 nM, 625 nM, 2500 nM and 10,000 nM. The evaluation of binding kinetics was performed with Biacore T200 Evaluation Software 3.0. Both a 1:1 binding model as well as a two-state reaction model were tested. Figures were prepared in GraphPad Prism.

## Data availability

The atomic coordinate model for TG2/FN45 complex has been deposited in the PDB with accession code 30UC. The corresponding CryoEM map is deposited in the EMDB with the accession code: EMD-58048.

## Supporting information

This article contains supporting information. Tables S1–S4 and figures S1–S8.

## Conflict of interest

The authors declare that they have no conflicts of interest with the contents of this article.
