# Supplementary material for: Cryo-EM structure of the complex between transglutaminase 2 and the 45 kDa domain of fibronectin
Source: J Biol Chem. 2026 Jul 13;302(9):113326. doi: 10.1016/j.jbc.2026.113326 (PMC13485644; doi:10.1016/j.jbc.2026.113326)
Supplement: Supplementary file 1 [file mmc1.pdf]

Supporting information

Cryo-EM structure of the complex between transglutaminase 2 and the 45 kDa domain of fibronectin

Julie E. Heggelund[a,b], Ahmad Ali-Ahmad[c,d], Marie K. Johannesen[a,b], Nikolina Sekulić[c,d] and Ludvig M. Sollid[a,b]

a. Norwegian Coeliac Disease Research Centre, Institute of Clinical Medicine, University of Oslo, Oslo, Norway.
b. Department of Immunology, Oslo University Hospital - Rikshospitalet, Oslo, Norway
c. Norwegian Centre for Molecular Biosciences and Medicine (NCMBM), Nordic EMBL Partnership, Faculty of Medicine, University of Oslo, Oslo, Norway
d. Department of Molecular Medicine, Institute of Basic Medical Sciences, Faculty of Medicine, University of Oslo, Oslo, Norway

Table S1: LC-MS/MS analysis: detected peptides of FN45

Uniprot Accession number	FN Module	FN Start	FN End	Peptide	Intensity	MS/MS Count
P02751	I-1	58	67	HYQINQQWER	50470	1
P02751	I-1	68	83	TYLGNALVCTCYGGSR	194170	1
P02751	I-1 + I-2	84	107	GFNCESKPEAEETCFDKYTGNTYR	39999	1
P02751	I-6	291	324	AAVYQPQPHPQPPPYGHCVTDSGVVYSVGMQWLK	95191000	11
P02751	II-1	370	379	TFYSCTTEGR	52526000	14
P02751	II-1	370	397	TFYSCTTEGRQDGHLWCSTTSNYEQDQK	33474000	5
P02751	II-1	380	397	QDGHLWCSTTSNYEQDQK	97156000	11
P02751	II-1	380	411	QDGHLWCSTTSNYEQDQKYSFCTDHTVLVQTR	6526300	3
P02751	II-1	398	411	YSFCTDHTVLVQTR	68563000	5
P02751	II-2	440	457	RDNMKWCGTTQNYDADQK	236480	1
P02751	II-2	441	457	DNMKWCGTTQNYDADQK	5413000	3
P02751	II-2	445	457	WCGTTQNYDADQK	42735000	4
P02751	II-2 + I-7	458	479	FGFCPMAAHEEICTTNEGVMYR	160890000	15
P02751	I-7	480	486	IGDQWDK	887250	1
P02751	I-7	480	495	IGDQWDKQHDMGHMMR	9226700	10
P02751	I-7	496	515	CTCVGNGRGEWTCIAYSQLR	1158400	1
P02751	I-7	504	515	GEWTCIAYSQLR	39524000	5
P02751	I-8	516	533	DQCIVDDITYNVNDTFHK	150930	1
P02751	I-9	551	578	GRWKCDPVDQCQDSETGTFYQIGDSWEK	480750	1
P02751	I-9	553	578	WKCDPVDQCQDSETGTFYQIGDSWEK	11345000	3
P02751	I-9	555	578	CDPVDQCQDSETGTFYQIGDSWEK	11360000	3
P02751	I-9	555	584	CDPVDQCQDSETGTFYQIGDSWEKYVHGVR	50860	1
P02751	I-9	585	592	YQCYCYGR	46790000	14

Table S2: PISA analysis revealing buried residues and surface area of the interface between TG2 and FN45

	Fn all modules	Fn module I-8	Fn module I-7	Fn module II-2
TG2 buried residues	47	23	21	8
Fn buried residues	44	20	21	3
H-bonds	21	15	4	2
Salt bridges	9	4	4	1
TG2 buried area (Å)	1434.7	726.3	622.7	92.7
Fn buried area (Å)	1442.8	684.9	654.6	113.5
CSS	0.95			
ΔG (kcal/mol)	-9.0	-2.8	-6.8	0.5
CSS= Complex formation significance score. ΔG= change of Gibbs free energy upon complex formation				

Table S3: Buried surface area of TG2 residues and ΔG

Close contact with module I_8 (516-558)				
**TG2 residue**	**H/S**	**ASA**	**BSA**	**ΔG (kcal/mol)**
GLU3		114.28	0.50	0.01
GLU4		43.45	34.56	0.37
LEU5		3.99	2.82	-0.03
VAL6		72.92	49.90	0.80
SER56	H	69.50	24.67	-0.03
SER60		21.90	15.07	0.09
VAL62		19.22	1.67	0.03
GLN69		102.05	0.61	-0.01
LYS74	S	85.16	37.69	0.11
ALA75		7.90	0.37	0.00
ARG76	HS	142.61	54.45	-0.78
SER118		26.31	17.20	0.10
LEU119		0.69	0.37	0.00
GLU120	H	62.26	50.77	-0.25
SER122		45.41	20.64	0.09
GLY124	H	66.70	28.03	0.10
TYR125	H	214.34	142.13	1.11
GLN126		70.76	49.27	0.18
GLY127	H	38.16	38.16	-0.11
SER128		34.81	34.81	0.11
SER129	H	88.71	81.47	0.10
PHE130		45.94	27.88	0.37
VAL131		100.98	13.23	0.21
**Close contact with module I_7** (468-511 + loop 512-515)				
GLU29		171.43	6.87	-0.08
LYS30		52.33	8.18	0.06
GLY64		32.92	5.07	-0.01
PRO65		91.08	70.16	0.79
LEU114		89.52	42.69	0.68
ARG116	HS	122.07	54.68	-0.48
SER129		88.71	4.05	-0.05
VAL131		100.98	30.62	0.49
HIS134	HS	82.76	59.23	0.06
ASP191		64.59	30.53	-0.13
ILE195		50.89	24.46	0.39
ASP198		61.61	28.44	0.05
VAL199		39.41	28.69	0.46
PHE203		39.39	34.22	0.53
LEU204	H	164.23	101.89	1.25
LYS205		152.78	6.21	-0.03
ALA207		29.35	6.87	0.11
GLN234		49.06	16.37	-0.09
LYS265	S	89.63	22.71	0.10
GLY268		57.06	26.98	0.30

CYS269		13.75	13.75	0.47
**Close contact with module II_2** (415-463 + loop 409-414)				
VAL199		39.41	1.48	-0.02
PRO201		65.11	0.84	0.01
LEU204		164.23	16.56	0.27
ASN231		16.19	4.89	-0.06
ASP232	H	108.88	10.21	-0.10
ASP233	HS	97.80	29.91	-0.04
GLN234		49.06	28.24	0.20
GLN270		77.29	0.62	-0.01
H= hydrogen bond, S= salt bridge, ASA= accessible surface area, BSA=buried surface area, ΔG= change of Gibbs free energy upon complex formation				

Table S4: Electrostatic interactions of TG2 with FN45

TG2 residue	Distance (Å)	FN45 residue	H / S
**Binding to module I_8** (516-558)			
SER56[OG]	3.26	GLU536[OE2]	H
LYS74[NZ]	3.66	ASP522[OD2]	S
ARG76[NH1]	3.5	ASP521[OD2]	S
ARG76[NH2]	3.48	ASP521[OD2]	S
ARG76[NE]	3.66	ASP522[OD1]	S
ARG76[NH1]	3.5	ASP521[OD2]	H
ARG76[NH2]	3.48	ASP521[OD2]	H
GLU120[OE1]	3.83	ASP521[N]	H
GLU120[OE2]	3.59	ASP522[N]	H
GLY124[O]	3.55	LYS533[NZ]	H
TYR125[O]	3.04	LYS533[NZ]	H
TYR125[OH]	3.39	HIS539[NE2]	H
GLY127[O]	2.93	TRP553[N]	H
GLY127[N]	2.99	TRP553[O]	H
SER129[OG]	2.87	ILE519[N]	H
SER129[OG]	3.12	TRP553[NE1]	H
SER129[OG]	2.84	ILE519[O]	H
SER129[N]	3.59	ARG550[O]	H
SER129[N]	3.37	GLY551[O]	H
**Binding to module I_7** (468-511 + loop 512-515)			
ARG116[NH1]	3.34	ASP485[OD2]	S
ARG116[NH1]	3.37	TYR511[OH]	H
ARG116[NH1]	3.34	ASP485[OD2]	H
HIS134[NE2]	3.42	ASP485[OD1]	S
HIS134[NE2]	3.96	ASP485[OD2]	S
**Binding to module II_2** (415-463 + loop 409-414)			
ASP232[O]	3.25	ARG440[NH1]	H
ASP233[OD2]	3.72	ARG440[NH1]	S
ASP233[OD2]	3.72	ARG440[NH1]	H
H= hydrogen bond, S= salt bridge			

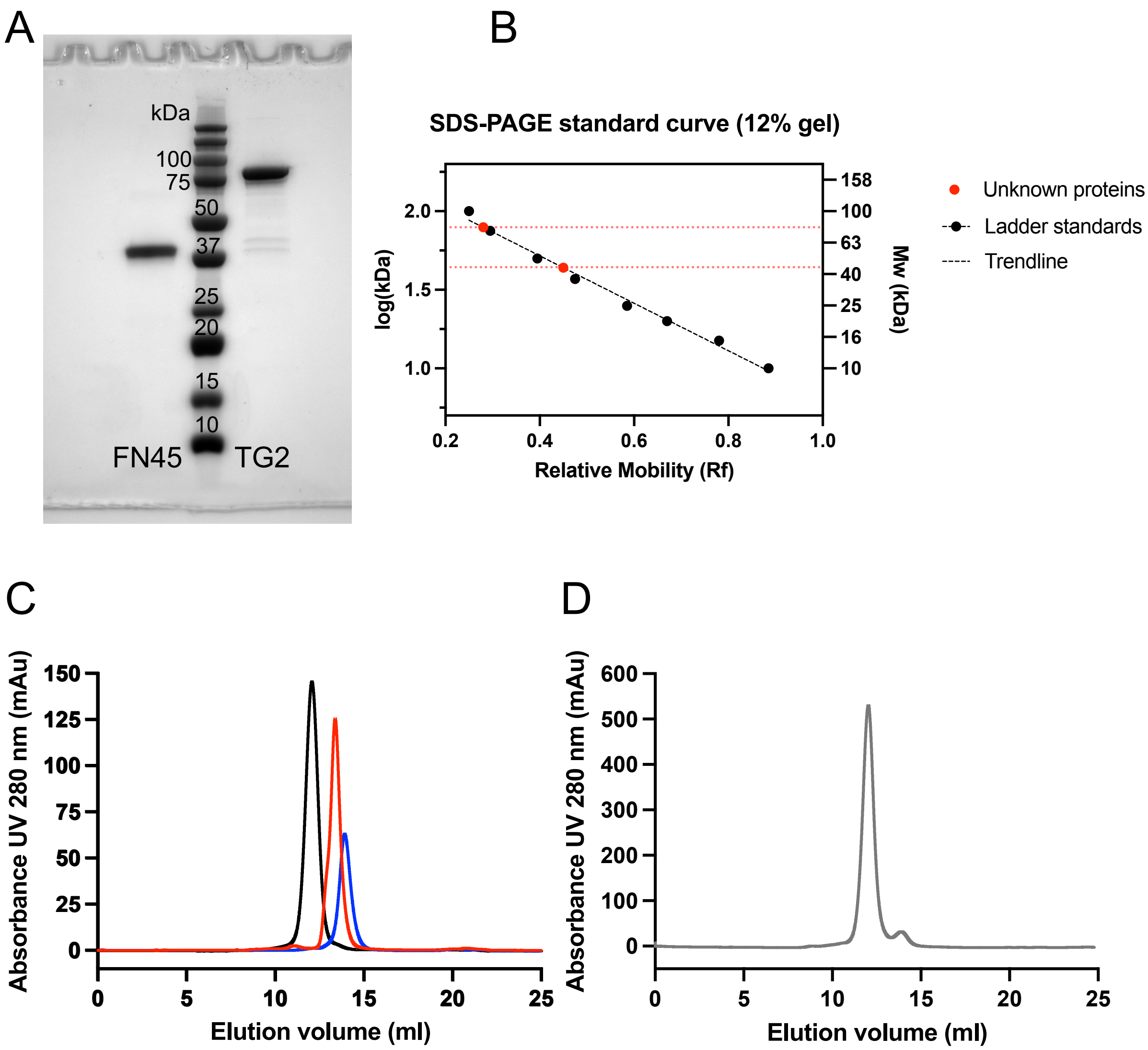

Figure S1. Characterization of TG2 and FN45 proteins and their complex. A. SDS-PAGE of TG2 and FN45 before they were mixed for complex formation. A 12% Tris-glycine gel was loaded with 2 mg each of FN45 and TG2 in separate wells. Molecular weight standards are in the middle lane. **B.** Standard curve of the SDS-PAGE molecular weight standards shown in S1A. Red dots and red lines indicate TG2 and FN45 which were calculated to be 79 kDa and 44 kDa, respectively. **C.** Overlay of chromatograms from three size exclusion chromatography runs. Black line: TG2+FN45 complex. Red line: TG2. Blue line: FN45. mAu: milli-absorbance units. **D.** Chromatogram of the final TG2–FN45 complex run used for cryo-EM. Only the middle fraction of the peak with the complex was collected for analysis.

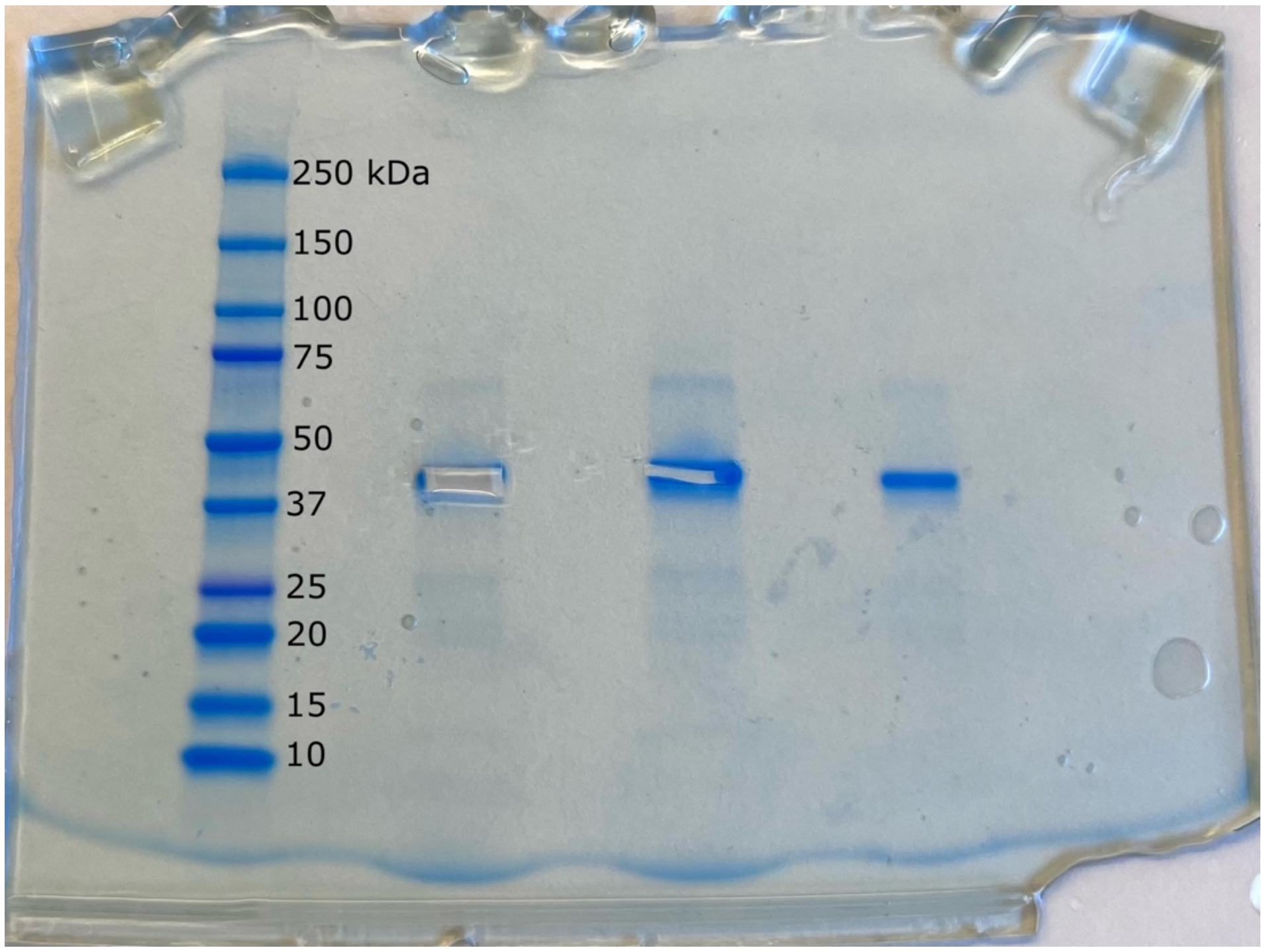

Figure S2. SDS PAGE analysis of FN45. A 4-20% Tris-glycine gel with FN45 loaded in three different amounts (2.25 µg, 4.5 µg and 0.6 µg). Two of the bands were excised, and the middle band was chosen for analysis by LC-MS/MS.

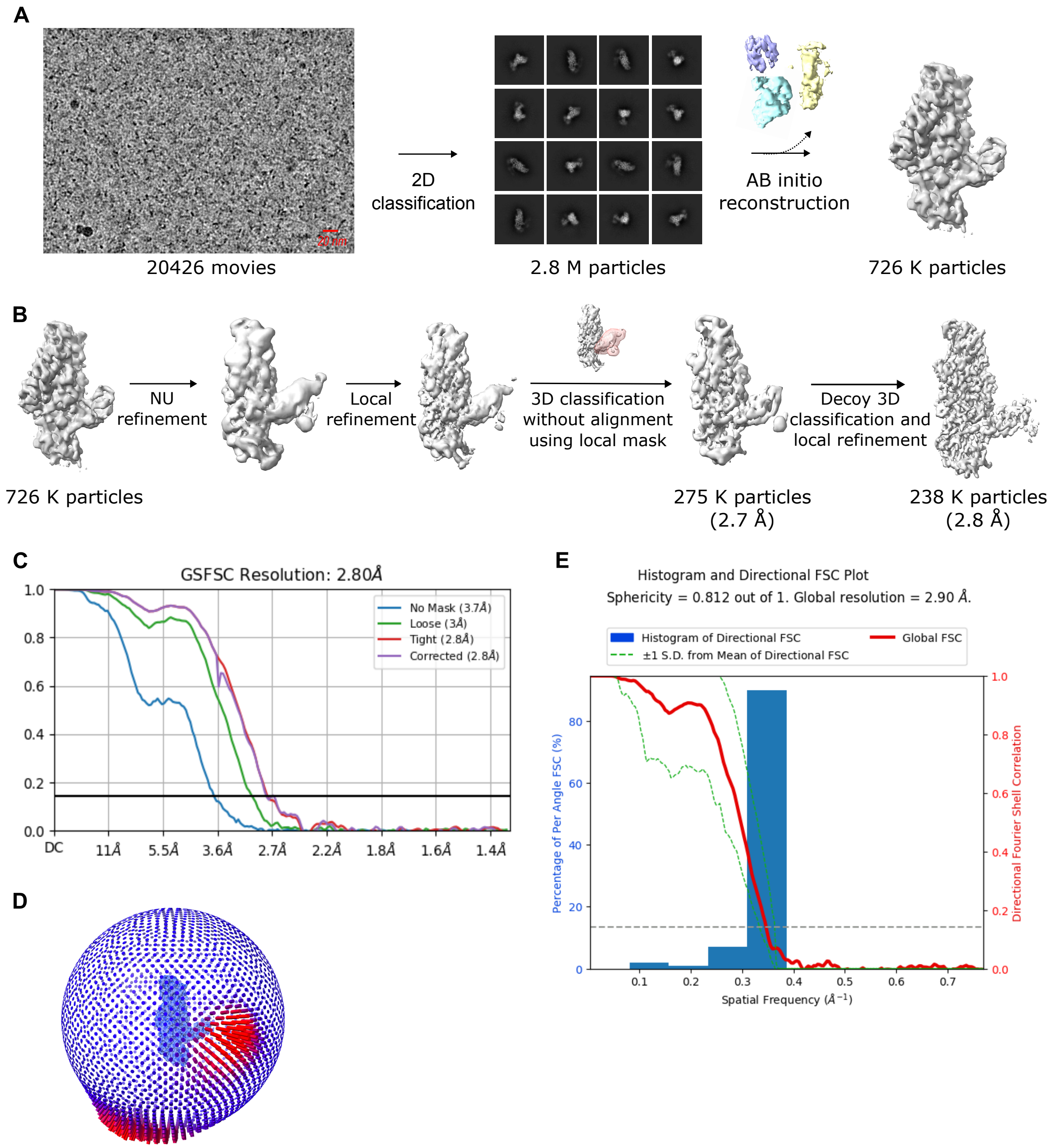

Figure S3. Workflow of image processing and 3D construction of cryo-EM images of TG2-FN45 complex. A. From left to right: Representative cryo-EM micrograph, subset of selected 2D class averages and the reference map for the complex generated using *ab initio* reconstruction. Discarded classes from ab initio reconstruction are shown in yellow, cyan and purple. **B.** Flowchart of cryoEM data processing for TG2-FN45 complex. The *ab initio* reconstruction was followed by NU and local refinement before running 3D classification using a local mask covering FN45 density. The particles from the class with more resolved FN45 density were further processed using 3D decoy classification and local refinement. **C.** The FSC curve of the final map resulting from local refinement. **D.** Euler angle distribution of particles used in the 3D reconstruction of the final

map. **E.** Histogram of directional FSC plot showing mainly uniform resolution in all directions and a sphericity of 0.812.

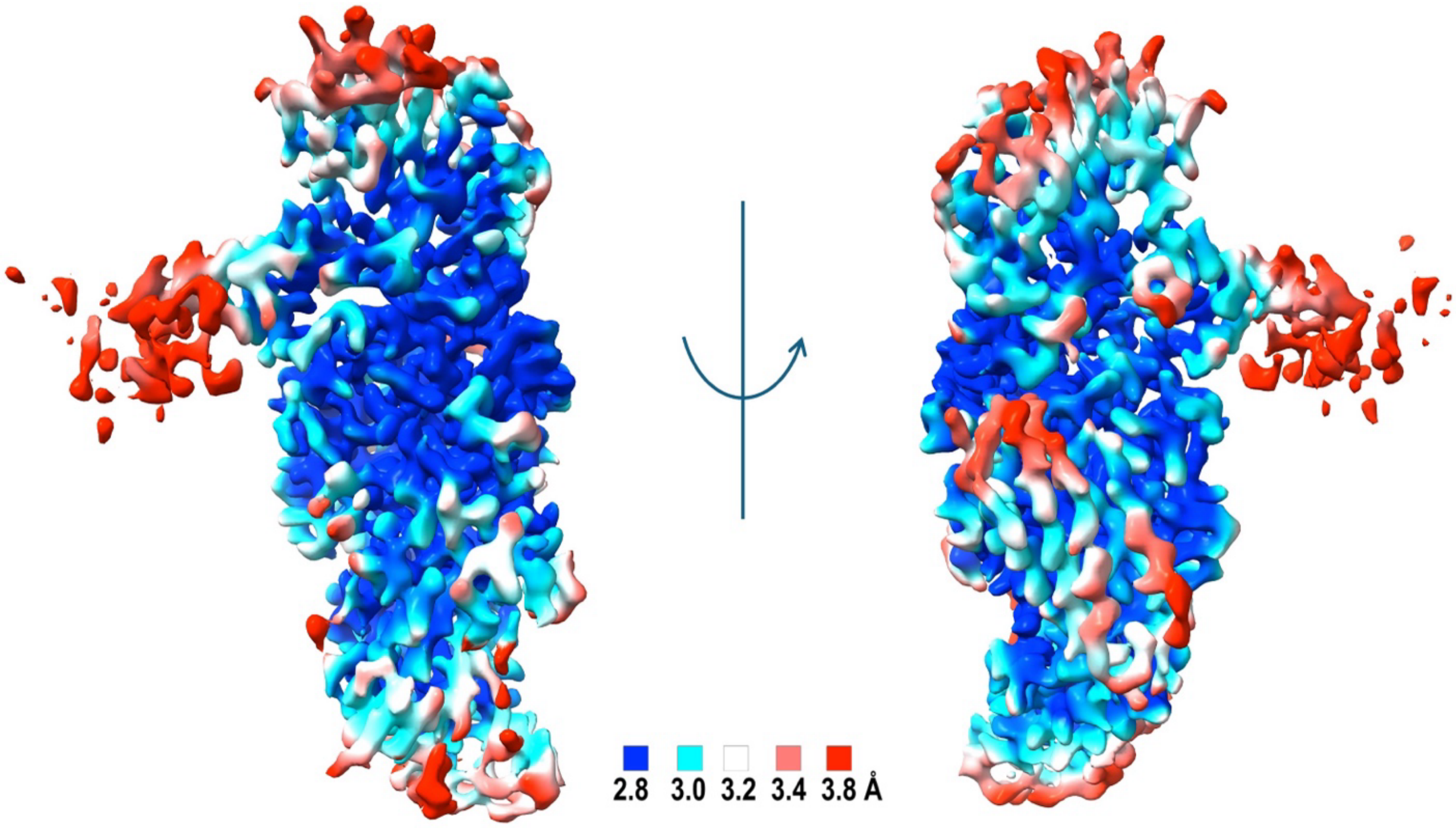

Figure S4. Local resolution map of TG2-FN45 complex. The color coding represents resolution at the different areas of the TG2-FN45 complex.

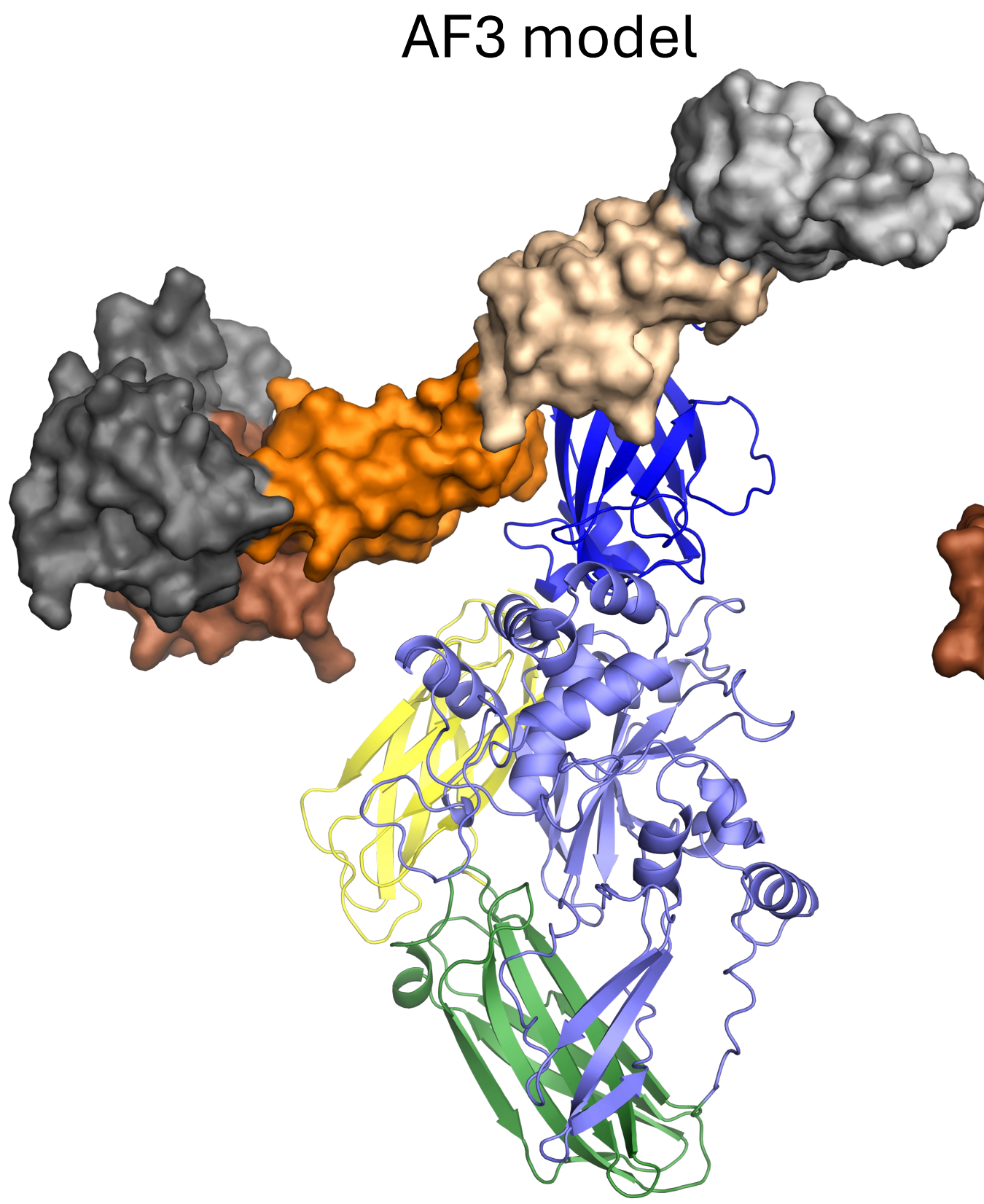

Figure S5. Comparison of AlphaFold3 and cryo-EM models of the interaction between TG2 and FN45. TG2 is shown in cartoon representation with the N-terminal domain in blue, the core domain in light blue, the C1 domain in green and the C2 domain in yellow. Glycosylation of FN45 is shown as blue sticks. FN45 is shown in surface representation with the I_9 module in light grey, the I_8 module in wheat, the I_7 module in orange, the II_2 module in brown and the II_1 and I_6 in dark grey. The I_9, II_1 and I_6 modules were not possible to place in the cryo-EM model due to poor local resolution.

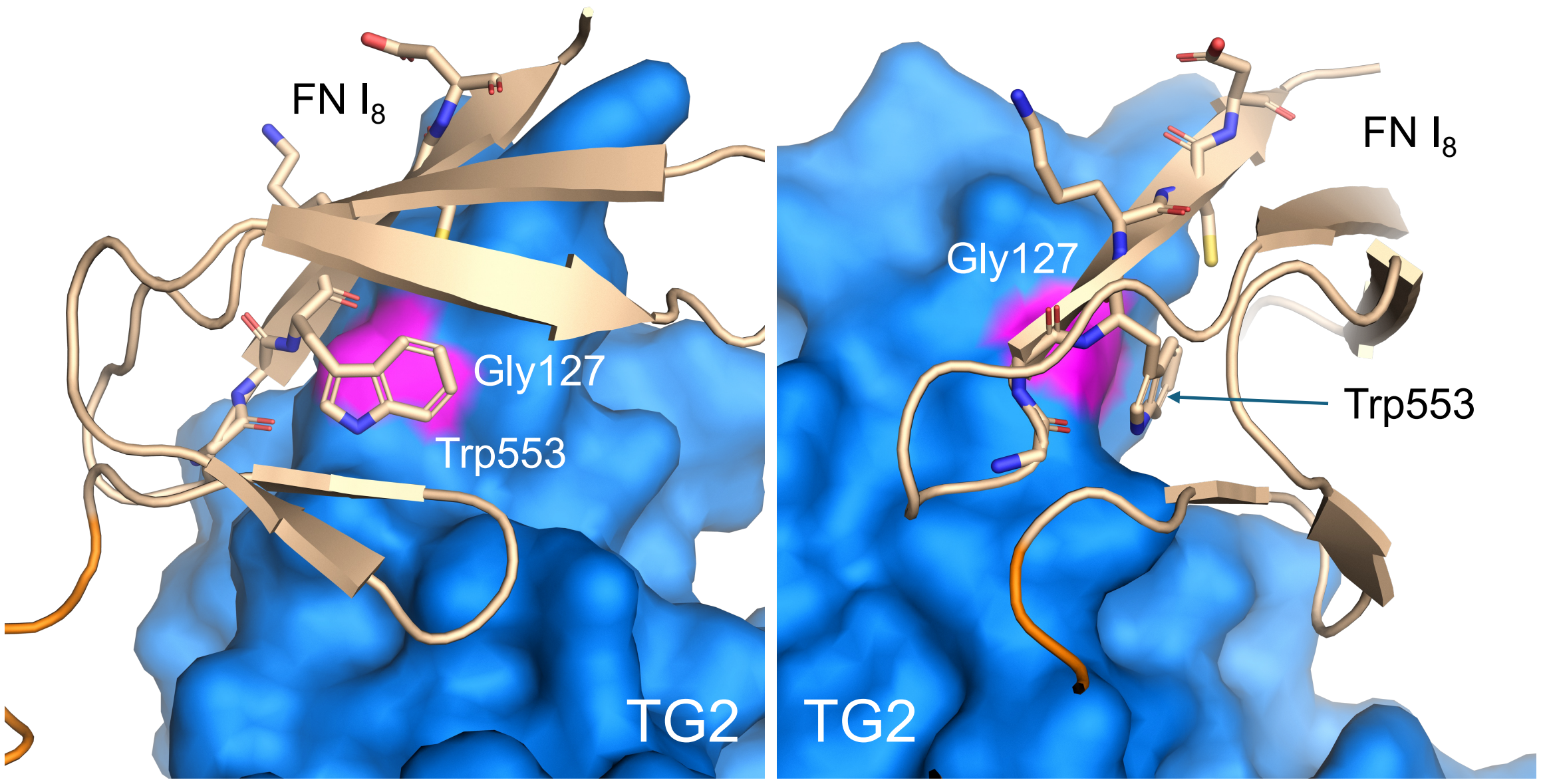

Figure S6. Cavity in TG2 around residue 127 at the binding site of FN I_8. TG2 is shown in blue surface representation with Gly127 indicated in magenta. FN45 is shown in cartoon representation with residues of interest shown in sticks. Trp553 of FN I_8 is tightly bound in a shallow groove in the N-terminal domain of TG2 close to Gly127.

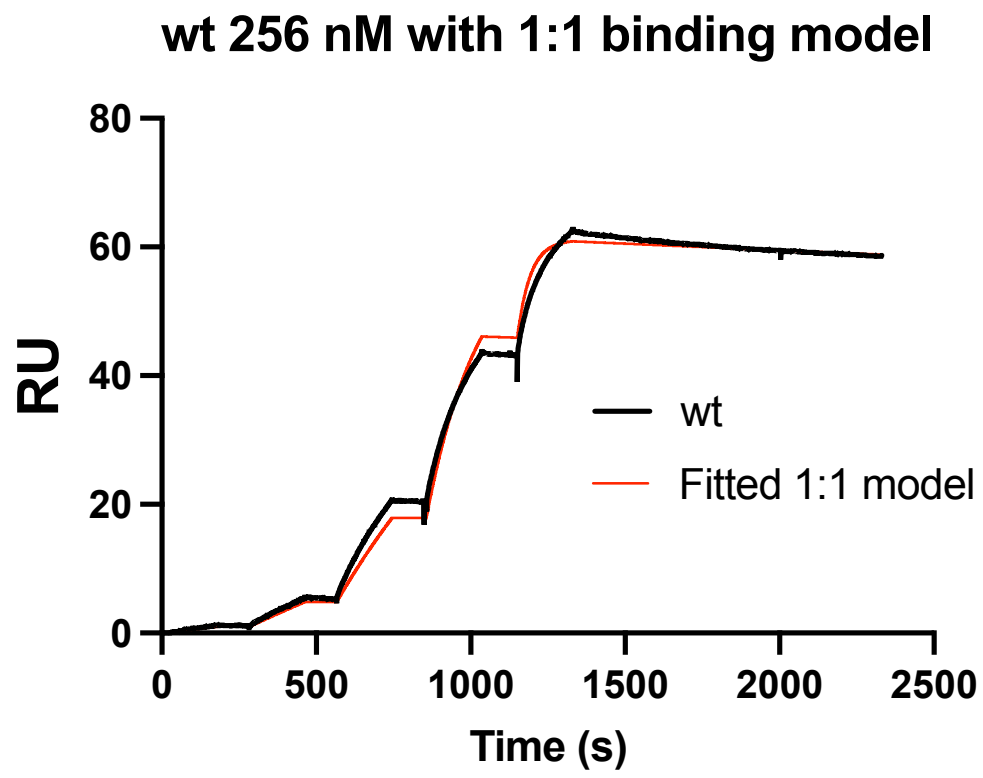

Chi^2: 1.85 RU^2
ka: 92320 $M^{-1}s^{-1}$
kd: 0.00003475 s^{-1}
KD: 0.38 nM

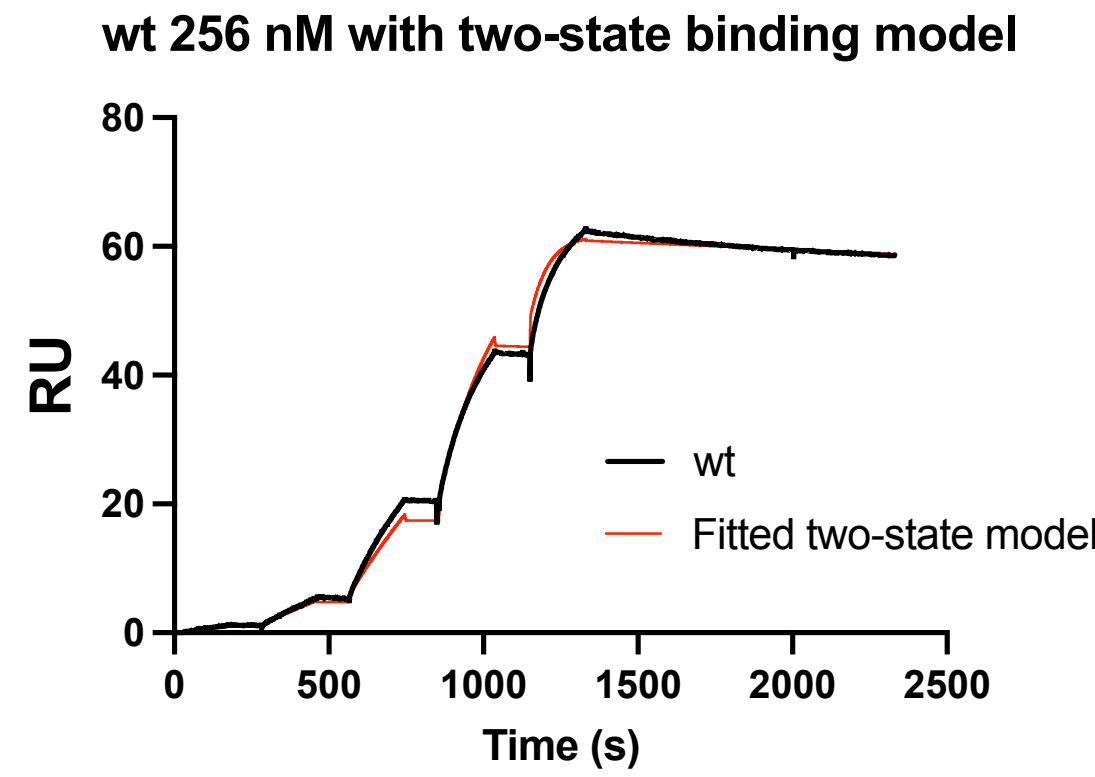

Chi^2: 1.47 RU^2
ka1: 1387000 $M^{-1}s^{-1}$
kd1: 0.9223 s^{-1}
ka2: 0.06392 s^{-1}
kd2: 0.0000372 s^{-1}
KD: 0.39 nM

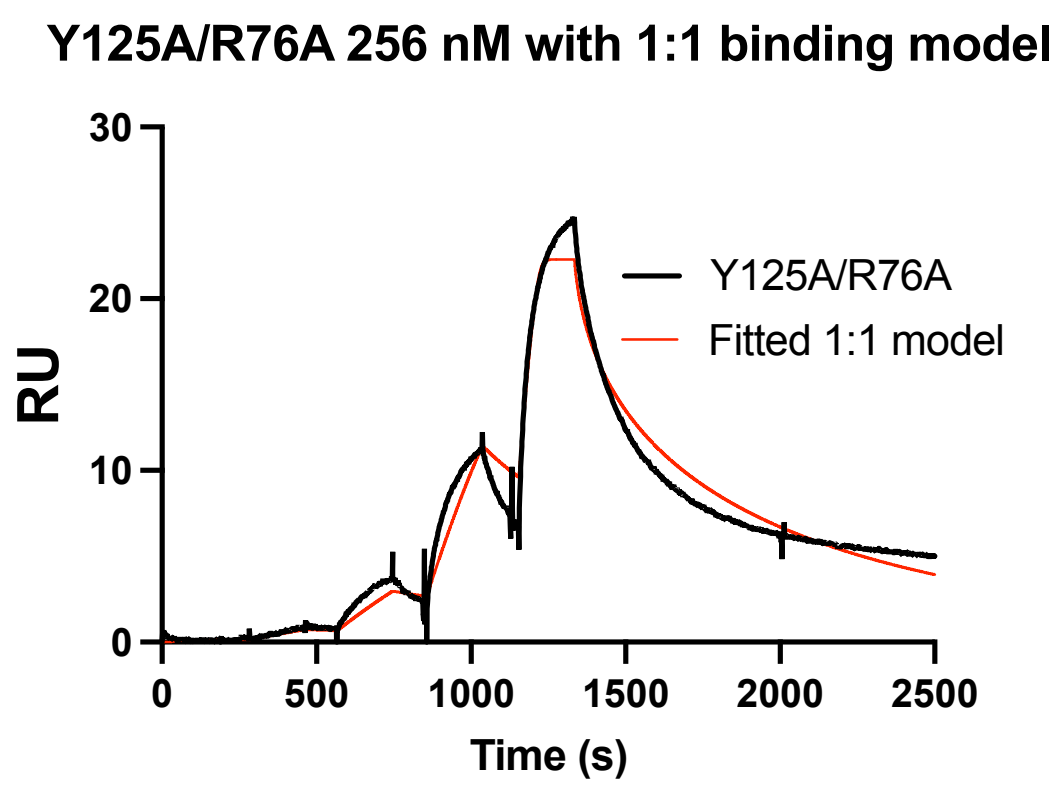

Chi^2: 1.12 RU^2
ka: 1062000000 $M^{-1}s^{-1}$
kd: 23.78 s^{-1}
KD: 22.4 nM

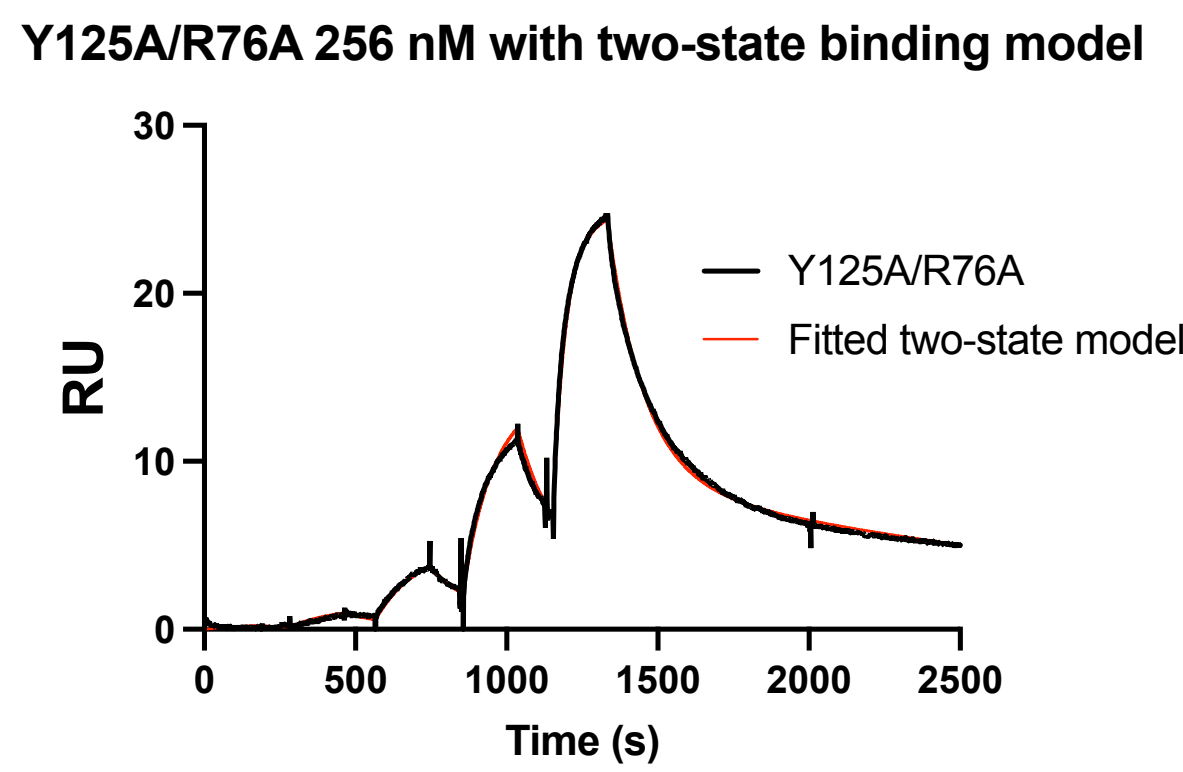

Chi^2: 0.116 RU^2
ka1: 71590 $M^{-1}s^{-1}$
kd1: 0.007361 s^{-1}
ka2: 0.001133 s^{-1}
kd2: 0.00058 s^{-1}
KD: 34.8 nM

Figure S7. Evaluation of representative SPR sensorgrams. The data were analyzed using the 1:1 binding model as well as the two-state binding model. For the TG2 variants, represented here by Y125A/R76A, the employment of the two-state model improved the chi^2 values drastically, and led to an overall much better fit. For the wt protein, the two-state model resulted in an injection spike and a corresponding extreme value of kd1 of 0.92 s^{-1}. Based on these observations, the two-state model was rejected for the wt protein.

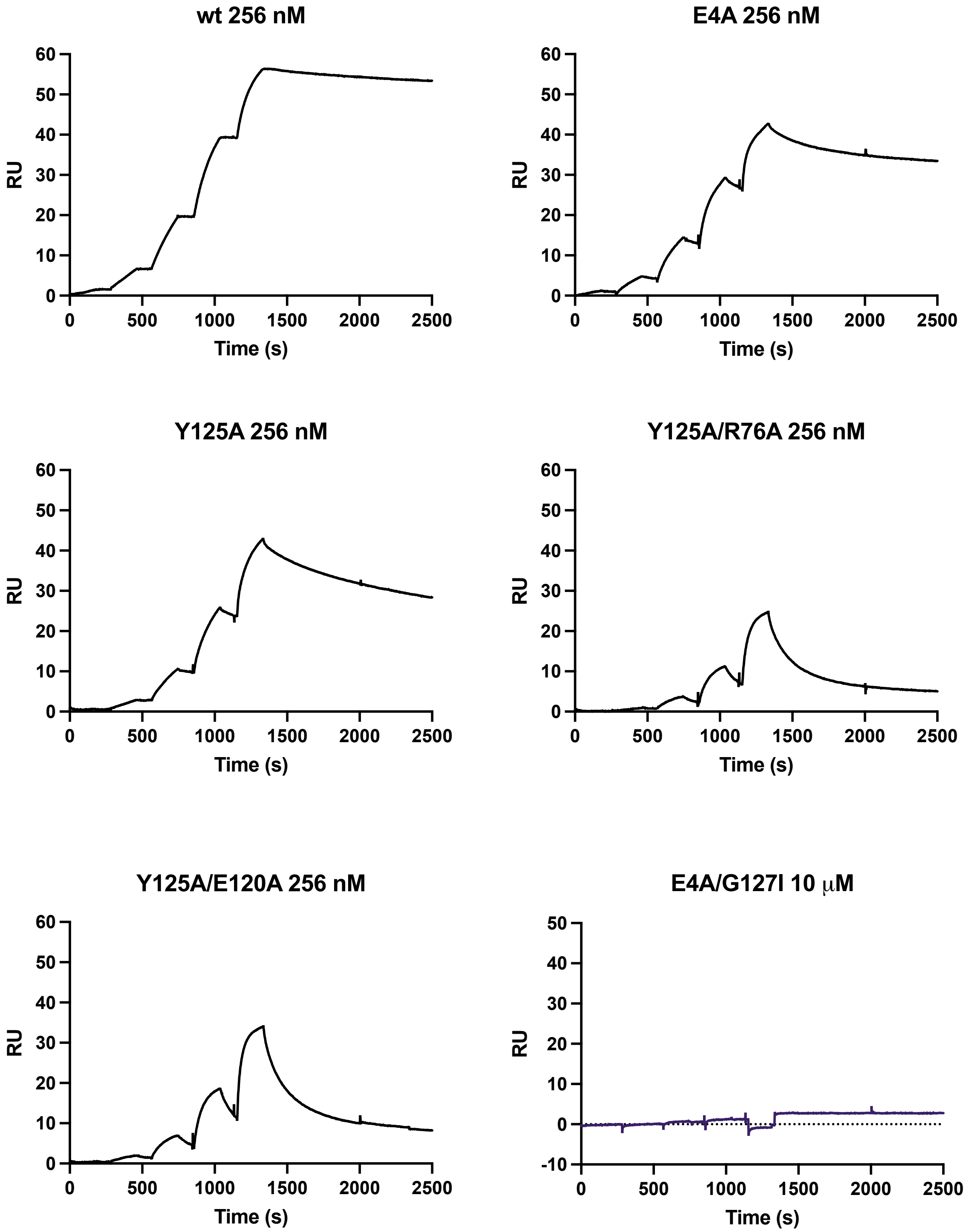

Figure S8. SPR analysis of binding of TG2 variants to FN45. Representative SPR sensorgrams of TG2 and variants binding to FN45. The single cycle experiments were performed at TG2 concentrations 1, 4, 16, 64 and 256 nM, except the E4A/G127I variant, which was run at concentrations 39, 156, 625, 2500 and 10000 nM. The highest concentration of E4A/G127I resulted in significant buffer mismatch as can be seen by the dip in the sensorgram at time point 1200 s.
